# PReS-FINAL-2312: Cogan's syndrome: a diagnostic challenge. Report and review of the literature

**DOI:** 10.1186/1546-0096-11-S2-P302

**Published:** 2013-12-05

**Authors:** A Lazareva, ZD Davidsone, R Santere, S Kuske, V Stanevicha

**Affiliations:** 1 Pedaitrics, Children's University Hospital, Riga, Latvia; 2 Pediatrics, Children's University Hospital, Riga, Latvia; 3 Latvian Children's Hearing Center, Riga Eastern Clinical University Hospital, Riga, Latvia; 4 Pediatrics, Children's University Hospital, Riga, Latvia

## Introduction

Cogan's syndrome is a rare disorder of unknown origin characterized by inflammatory eye disease and vestibuloauditory symptoms. Usually syndrome affects young adults but cases in children have been reported. Typically, patients suffer from interstitial keratitis and sudden onset of tinnitus and hearing loss.

## Objectives

To review clinical characteristics of patients who develop Cogan's syndrome and review treatment possibilities.

## Methods

We describe 17 years old boy, previously healthy, who developed headaches, fever, lower leg weakness and pain, hypotension and conjuctivitis. Patient also developed mild stomatitis and during fever a couple of times patient developed maculopapular rash on his arms. After a month patient started to complain of vertigo, tinnitus and sudden hearing loss and after exclusion of acute and chronic infections and haematooncological disease treatment with steroids was started in addition with azathioprine. His tinnitus resolved after first steroid-pulse but hearing loss fully restored only in one ear.

## Results

Cogan's syndrome is a rare autoimmune vasculitis characterized by ocular and vestibuloauditory dysfunction, mostly described in young Caucasian patients of either sex. Etiology is unknown but infection and autoimmunity plays role in the development of Cogan's syndrome. In addition to ocular and vestibuloauditory involvement, numerous systemic manifestations have been reported, including aortitits, necrotizing vasculitis, constitutional features, gastrointestinal and neurological manifestations. There is no specific laboratory findings of Cogan's syndrome. Disease course is variable. Treatment can include glucocorticoids and in case of failure, other immunosuppressive drugs can be used.

## Conclusion

Despite its rarity, Cogan's syndrome is an important condition to recognize because early treatment may prevent the onset of profound deafness in children.

## Disclosure of interest

None declared.

